# Unraveling the role of METTL14 in metabolic dysfunction-associated fatty liver disease: insights and therapeutic implications

**DOI:** 10.1038/s41392-024-01837-w

**Published:** 2024-04-29

**Authors:** So Jung Kim, Jeongeun Hyun

**Affiliations:** 1 Institute of Tissue Regeneration Engineering (ITREN), Dankook University, Cheonan, Republic of Korea; 2 Department of Nanobiomedical Science and BK21 PLUS NBM Global Research Center for Regenerative Medicine, Dankook University, Cheonan, Republic of Korea; 3 Mechanobiology Dental Medicine Research Center, College of Dentistry, Dankook University, Cheonan, Republic of Korea

**Keywords:** Epigenetics, Innate immunity

The recent article published in *Signal Transduction and Targeted Therapy* sheds light on the significance of N6-methyladenosine (m6A) methyltransferase-like protein METTL14 in mitigating the progression of metabolic dysfunction-associated fatty liver disease (MAFLD).^1^ In this study, Wang et al. elucidated the downregulation of METTL14 in hepatocytes from both MAFLD patients and high-fat diet (HFD)-induced MAFLD mouse models, underscoring its pivotal role in maintaining hepatic lipid and redox homeostasis in normal livers. Dysregulation of METTL14 emerges as a contributing factor to MAFLD development.

The increasing global prevalence of MAFLD poses a significant clinical and economic burden. MAFLD represents a multifaceted condition influenced by diverse genetic and environmental factors, leading to varied disease presentations and complicating effective drug development.^2^ Despite recent advancements such as the accelerated approval of Rezdiffra (Resmetirom) by the Food and Drug Administration for noncirrhotic metabolic dysfunction-associated steatohepatitis (MASH) treatment, the complexity of MAFLD necessitates continued exploration of precise mechanisms and identification of druggable targets.

The methyltransferase-like (METTL) protein family, characterized by *S*-adenosyl methionine-binding domains, is known for its diverse methyltransferase activities, acting on a wide spectrum of substrates that include nucleotides, small molecules, and proteins. Among the diverse activities, m6A modifications mediated by METTL proteins in the nucleus play a crucial role in post-transcriptional RNA regulation, impacting mRNA splicing, degradation, translation, and stability.^3^ Notably, m6A modifications written by METTL proteins are subsequently recognized by readers such as the YT521-B homology (YTH) domain family (YTHDF), mainly in the cytoplasm. Additionally, other m6A RNA-binding proteins, such as YTH domain containing (YTHDC), can recognize m6A modifications in either cytoplasm or nucleus. While previous studies have highlighted the significance of METTL3 and METTL14 in MAFLD, conflicting evidence warrants further investigation into their roles and therapeutic potential.

Excessive lipid accumulation is a hallmark of MAFLD, and the progressed form of MAFLD, MASH, is characterized by inflammation and often accompanies fibrosis.^2^ Through comprehensive metabolomics analysis and biochemical assays, Wang et al. demonstrated the m6A-dependent translational regulation of liver-type glutaminase (GLS2) by METTL14 and YTHDF1, defining the sites of m6A methylation in *GLS2* mRNA. In detail, manipulation of METTL14 expression affected GLS2 at the protein level but not at the mRNA level, independent of proteasome activity, indicating that METTL14 affects mRNA translation but not mRNA and protein stability of GLS2. GLS2, a periportal hepatocyte enzyme catalyzing the conversion of glutamine to glutamate, exerts antioxidant functions by increasing glutathione levels and reducing reactive oxygen species (ROS) levels through increasing glutamate levels, a precursor of glutathione.^4^ Therefore, loss of METTL14 led to GLS2 deficiency, reducing antioxidant capacity. Liver injury and lipid accumulation occurred with increased ROS levels, exacerbating MAFLD progression in hepatocyte-specific *Mettl14*-knockout (KO) mice.

Moreover, the authors delved into the compositional changes of myeloid cells in MAFLD livers of *Mettl14*-KO mice using single-cell RNA sequencing, recognizing the pivotal role of macrophages in the immune microenvironment with amplified oxidative stress and inflammation. Specifically, Wang et al. focused on elucidating the role of *Cx3cr1*^+^*Ccr2*^+^ monocyte-derived macrophages (Mo-macs), the proportion of which significantly increased in *Mettl14*-KO livers compared to *Mettl14*-wild type livers. They consistently found a high accumulation of *Cx3cr1*^+^*Ccr2*^+^ Mo-macs in the livers of HFD-fed mice. Interestingly, *Cx3cr1*^+^*Ccr2*^+^ Mo-macs emerged at the late stage of the developmental trajectory among Mo-macs compartments, suggesting that they are highly differentiated and exhibit specific behaviors and functions. Prompted by these observations, the authors investigated the role of this particular subtype of macrophages in the progression of MAFLD and elucidated the mechanisms underlying the recruitment of these cells into MAFLD livers.

Among the top 20 upregulated genes in *Cx3cr1*^+^*Ccr2*^+^ Mo-macs compared to *Cx3cr1*^*-*^*Ccr2*^-^ Mo-macs, four S100 calcium-binding protein A (*S100A*) genes, including *S100a4*, *S100a6*, *S100a10*, and *S100a11*, are identified. Additionally, Wang et al. found that *Cx3cr1*^+^*Ccr2*^+^ Mo-macs co-express MyD88 (*Cx3cr1*^+^*Ccr2*^+^*MyD88*^+^), a protein interacting with toll-like receptors (TLRs) in response to damage-associated molecular patterns, such as 8-hydroxy-2’-deoxyguanosine (8-OHdG), in the livers of *Mettl14*-KO and HFD-fed mice, as well as in macrophages differentiated from THP-1 monocytes. MyD88 plays crucial roles in innate immune responses and is known to induce the nuclear factor κB (NF-κB) signaling pathway, thereby promoting the expression of pro-inflammatory cytokines and S100A proteins. These S100A proteins, in turn, amplify TLR-mediated signaling and activate hepatic stellate cells, ultimately promoting liver fibrosis.^5^ Finally, the authors demonstrated that *Cx3cr1*^+^*Ccr2*^+^*MyD88*^+^ Mo-macs produce S100A proteins, transcriptionally promoted by NF-κB signaling downstream of CX3CR1. Of note, the authors mentioned that the mechanism of METTL14/GLS2 deficiency-mediated liver fibrosis illustrated in this study is specific to MASH-related fibrosis and may not be applicable to hepatitis B virus (HBV)-induced fibrosis. However, more comprehensive studies are needed to draw firm conclusions, as their evaluation of this mechanism in HBV-induced fibrosis relied solely on immunostaining in liver cancer patient tissues with HBV.

Nevertheless, this study holds promising clinical implications as it proposes potential therapeutic strategies to alleviate the progression of MAFLD, and importantly, it experimentally validates their effectiveness. Firstly, Wang et al. restored METTL14 function in *Mettl14*-KO mice by employing a *Mettl14*-overexpressing adeno-associated virus (AAV) vector. It significantly alleviated liver injury, inflammation, and fibrosis by increasing GLS2 expression and reducing ROS and 8-OHdG levels, and the number of S100A4-expressing macrophages. Secondly, similar favorable outcomes were achieved following GLS2 restoration using an AAV vector in *Mettl14*-KO mice and in a MASH mouse model induced with a Western diet plus carbon tetrachloride treatment. These METTL14 or GLS2 restoration approaches aimed to mitigate hepatocyte oxidative stress at early stages of MAFLD progression. Thirdly, the authors proposed targeting *Cx3cr1*^+^*Ccr2*^+^*MyD88*^+^ Mo-macs-mediated liver fibrosis, which showed improvement upon treatment with the MyD88 inhibitor, ST2825, in *Mettl14*-KO mice.

**Fig. 1:**
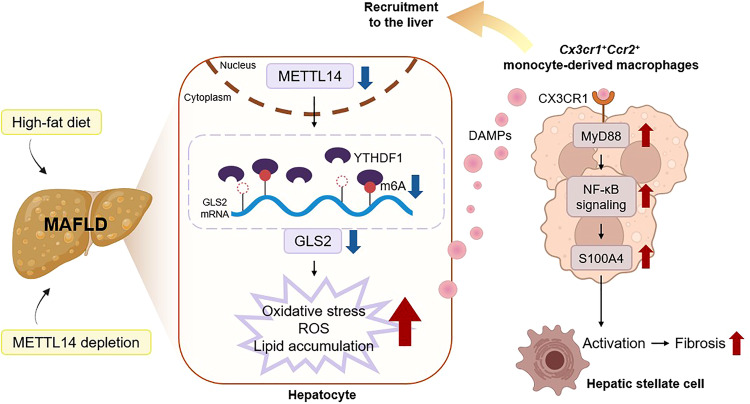
Schematic overview of METTL14 deficiency-mediated mechanism of MASLD progression. Hepatocyte METTL14 plays a crucial role in the pathogenesis of the metabolic dysfunction-associated fatty liver disease (MAFLD). In MAFLD induced by a high-fat diet or hepatocyte-specific *Mettl14* depletion, the METTL14-mediated m6A modification of *Gls2* mRNA is reduced, leading to decreased translation dependent on YTHDF1. This reduction in GLS2 abundance diminishes the antioxidant capacity of hepatocytes, resulting in elevated levels of reactive oxygen species (ROS), lipid accumulation, and oxidative stress. Hepatocyte damage caused by these processes releases damage-associated molecular patterns (DAMPs), which in turn recruit and activate CX3CR1/MyD88/NF-κB signaling in *Cx3cr1*^*+*^*Ccr2*^*+*^ monocyte-derived macrophages. Consequently, the pro-inflammatory and pro-fibrotic factor S100A4 is produced and released, contributing to the activation of hepatic stellate cells and the induction of liver fibrosis. Taken together, loss of METTL14 in hepatocytes triggers hepatocyte damage, inflammation, and fibrosis, characteristic features of metabolic dysfunction-associated steatohepatitis (MASH), thereby suggesting it as a potential therapeutic target for the treatment of MAFLD. The figure is created with Biorender.com

In summary, the findings presented by Wang et al. underscore the critical role of hepatocyte METTL14 in regulating GLS2 translation and subsequent ROS-mediated MAFLD progression. The oxidative stress condition provokes the inflammation and recruitment of *Cx3cr1*^+^*Ccr2*^+^*MyD88*^+^ Mo-macs that drive liver fibrosis via CX3CR1/MyD88/NF-κB/S100A pathway (Fig. 1). This study provides valuable insight into MAFLD pathogenesis, laying the groundwork for novel therapeutic approaches in its management.
